# Centering transgender and gender nonconforming people of color in the study of minority stress, transition-related factors, and mental health

**DOI:** 10.3389/fpubh.2026.1716010

**Published:** 2026-02-19

**Authors:** Michelle R. Dalton, Scott Secrest, Aline Araujo Hoffmann, Maria Nash, Divya Patel, Joanne Salas, M. Paz Galupo, Katie Heiden-Rootes

**Affiliations:** 1 Department of Family and Community Medicine, Saint Louis University School of Medicine, St. Louis, MO, United States; 2 School of Medicine, Saint Louis University School, St. Louis, MO, United States; 3 Advanced HEAlth Data (AHEAD) Research Institute, Saint Louis University School of Medicine, St. Louis, MO, United States; 4 Brown School of Social Work, Washington University in St. Louis, St. Louis, MO, United States

**Keywords:** gender congruence, gender affirming care, intersectionality, minority stress, nonbinary, POC, transgender

## Abstract

**Background:**

Transgender and gender non-conforming individuals (TGNC) people of color (POC) are exposed to a unique intersection of gender and racial-minority stressors. Distal (external) and proximal (internalized) stressors have been studied within the Gender Minority Stress Model, which outlays the adverse mental/physical health outcomes that often occur due to gender-minority stress. Few studies have examined the intersectionality of racial-minority related stress, mostly utilizing White TGNC samples, limiting generalizability to TGNC POC.

**Methods:**

We conducted an online cross-sectional survey (*n* = 149) TGNC POC U.S. adults who answered demographics, questions regarding transition experiences (e.g., gender-affirming care and identification documents), intersectional distal stressors (LGBT People of Color Microaggressions Scale), proximal stressors (The Gender Minority Stress and Resilience Measure), the Gender Congruence and Life Satisfaction Scale (GCLS), the Patient Health Questionnaire-9, and the Generalized Anxiety Disorder-7.

**Results:**

91.3% desired a legal name change and a gender marker change (98%). Less than 60% who wanted changes were able to obtain them, reporting negative outcomes using incongruent documents. Additionally, 78.2% of those who wanted gender affirmative hormone therapy were able to obtain it (*n* = 55). A path analysis using structural equation modeling revealed that distal stressors were both directly and indirectly, through proximal stressors, related to anxiety (direct: *β* = 0.42, 95% CI = 0.26, 0.58, indirect: *β* = 0.16, 95% CI = 0.07, 0.26) and depression (direct: *β* = 0.43, 95% C I = 0.26, 0.60, indirect: *β* = 0.14, 95% CI = 0.04, 0.23). Intersectional distal stress was not directly related to congruence but was indirectly related through proximal stressors (indirect effect: *β* = −0.16, 95% CI = −0.27, −0.05); Intersectional distal stress was not directly related to congruence but was indirectly related through proximal stressors (indirect effect: *β* = −0.16, 95% CI = −0.27, −0.05). Interpretation should be cautioned given low reliability of the GCLS.

**Conclusion:**

Findings emphasize intersectionality and internalization of those stressors on gender congruence, anxiety, and depression, highlighting the importance of culturally humble, accessible services. The high frequency of adverse experiences due to document incongruence highlights the importance of policies that reduce barriers. Future research should strengthen congruence measurement with longitudinal designs.

## 1 Introduction

Transgender and gender non-conforming (TGNC) individuals are exposed to gender minority stressors including negative societal and cultural experiences and messages about their gender identity or expression, from both from external, distal sources (i.e., media, law, family, or peers) or from internal, proximal sources (i.e., internalized transphobia) (1). Distal experiences due to being TGNC are pervasive and occur in a variety of contexts, including interpersonal relationships (2), the healthcare system (3), therapy system (4), and in the workplace (5). According to Gender Minority Stress Theory (1), TGNC individuals may internalize distal stress experiences through proximal processes (i.e., internalized transphobia), contributing to negative mental health outcomes and health disparities (6, 7). The Gender Minority Stress Model (1) describes how minority stress experiences predict adverse mental health and physical health outcomes, including cancer, cardiovascular disease, respiratory disease, and immune system dysregulation (8, 9).

### 1.1 Gender dysphoria and gender congruence

Another form of psychological distress experienced by TGNC individuals is gender dysphoria, or psychological distress due to the incongruence between an individual’s gender identity and their sex assigned at birth (10). Although TGNC experience gender incongruence due to the misalignment, not all TGNC report feeling gender dysphoric (11, 12) or a desire to medically transition (13, 14). They may pursue other avenues to achieve gender congruence, such as a changing their name, pronouns, and/or gender presentation (15–17). The inability to engage in social or medical transition can intensify experiences of psychological distress, including gender dysphoria. For instance, being unable to obtain gender-affirming care (GAC), legal name change, or gender marker change on official identification documents is related to increases in gender dysphoria (18, 19). Gender dysphoria is related to psychological distress and heightened self-harm for TGNC youth and adults (20). Access to GAC is also associated with lower levels of anxiety, depression, and gender dysphoria (21, 22).

Historically, gender dysphoria and gender incongruence have been defined through the medical model, with clinical professionals using clinical criteria (10) such as body-based distress due to gender incongruence to assign a gender dysphoria diagnosis, often to access GAC through a gatekeeping model (23). The conceptualization of gender dysphoria as an inherent outcome of gender incongruence has been heavily critiqued by community-based samples as being reductionistic, demonstrating that TGNC individuals may experience distress due to experiences of being TGNC in society, such as due to gendered expectations (24), body-based distress (25), and/or internal distress related to gender embodiment (26). Given that gender congruence may result in alternative experiences (i.e., gender euphoria or transgender joy) rather than consistent, diagnosable distress (27, 28) and the limitations of existing measures for gender congruence (29), we focus on gender congruence rather than diagnosable gender dysphoria throughout.

### 1.2 TGNC and intersectionality

Within the TGNC community, TGNC people of color (POC) (i.e., Black/African American, Indigenous, Asian/Asian American, Latinx, and other racial and ethnic minority groups) experience distal stressors due to both race (racial minority stress) and gender identity (gender minority stress), resulting in significantly higher levels of gender identity-based discrimination and violence than White TGNC individuals (30). TGNC POC individuals, particularly Black/African American, face reduced access to gender dysphoria diagnoses and GAC due to the racial economic inequities in the United States of America (USA) (31–34). TGNC POC experience greater barriers to care due to economic and insurance barriers resulting from systemic racism, with 63.3% of Black TGNC, 57.3% Latinx, 63.9% Native American, and 59.1% Asian TGNC experiencing limited access to GAC compared to 68.2% of White TGNC, which was related to heightened psychological distress for TGNC POC (35). Additionally, TGNC POC experience higher rates of medical complications when accessing GAC, longer hospital stays, and higher healthcare costs compared to their White TGNC counterparts (36). This is concerning, given that TGNC who experienced recent healthcare discrimination were two to three times more likely to delay preventative and routine medical care (18). Providers engaged in gatekeeping for GAC may add unnecessary barriers, such as requiring additional sessions of GAC mental health care prior to providing letters required by surgeons and some GAHT providers (35), despite clinical recommendations for informed consent (23). Distal stressors, including microaggressions and barriers to GAC, are related to increased psychological distress for TGNC POC (35). TGNC POC have 2.7 higher odds of depression compared to their cisgender peers (37), reporting the highest baseline levels of depression and anxiety compared to both White and cisgender peers (7). Additionally, suicidality rates of more than 50% are reported for TGNC POC, with the highest rates being among transgender women (38). These disparities are impacted by a variety of systemic and interpersonal factors, including barriers to healthcare access and mental health support, chronic stress, discrimination within healthcare, lack of culturally informed medical care, socioeconomic inequalities, and identity-based stigma (35, 39).

Research now demonstrates how living in an oppressive and an inequitable environment or experiencing violence related to gender identity contributes to gender dysphoria for TGNC people (19). This is heightened for TGNC POC who experience additional barriers to GAC, such as limited availability of specialized providers, financial constraints, and lack culturally relevant counseling for TGNC POC (40, 41). These disparities may discourage some from pursuing GAC and reinforce existing medical mistrust in racial minority communities (42). Despite this, few studies utilizing the Gender Minority Stress Model have explored the significant impact of intersectional distal stress (i.e., racial minority stressors) on gender congruence. Many studies utilizing the model have recruited mostly White samples, limiting its application to TGNC POC (43). Intersectionality theory serves as a lens to understand the interconnectedness of identity for those with multiple minority identities, such as TGNC POC (44, 45). The Gender Minority Stress Model has been critiqued for its narrow, additive approach (46, 47), highlighting the need for studies to include variables across several identity categories, rather than a narrow focus on only transphobia and/or heterosexism with TGNC POC (48). The use of intersectional measures for minority stressors rather than separate measures for distal and racial minority stressors represents one potential method of broadening the focus of the Gender Minority Stress Model, an approach taken within this study.

Thus, the purpose of our study is to further explore the utility of the Gender Minority Stress Model with TGNC POC in the U.S. Specifically, the first aim is to explore the impact of intersectional distal stress (i.e., LGBT racial microaggressions) and proximal stressors (including internalized transphobia, negative expectations for the future, and nondisclosure of identity) on anxiety, depression, and gender congruence for TGNC POC adults within the U.S. For Aim 1, we hypothesize that: (1) higher levels of intersectional distal stress and proximal stress will be related to higher levels of anxiety and depression and lower levels of gender congruence and (2) distal stressors will be indirectly related to outcomes (anxiety, depression, gender congruence) through proximal stressors. The second aim is to describe gender transition experiences, including gender marker, name change, and access to GAC. This study is relevant due to the high risk factors and racial/ethnic inequities TNGC POC individuals are exposed to and the impact on mental health, as better understanding of the stressors of TGNC POC would better equip healthcare systems to meet the needs of TGNC POC adults and support providers in delivering effective and affirming care.

## 2 Materials and methods

The study design is an online, cross-sectional survey using Qualtrics (Qualtrics, Provo, UT). Data were collected anonymously online during October of 2023 as part of a larger study on intersectional minority stressors for TGNC POC adults. The study received Institutional Review Board approval from Saint Louis University on September 21, 2023 (33572).

### 2.1 Participants and recruitment

To be included in the study, participants had to (a) self-identify as a gender identity that does not match their sex assigned at birth (e.g., transgender, nonbinary, or gender nonconforming), (b) identify as a race other than White, (c) be at least 18 years old, and (d) reside within the U.S. Following recommendations to increase data integrity with sexual and gender minority samples in online surveys (49), we excluded participants with outlier response times (defined as under 200 s based upon pilot testing the survey with research staff) or those who were missing substantial amounts of data (>20%). Removing participants who answered quickly or had a substantial amount of missing data was to reduce bias due to careless responding or fraudulent responses (50–52). Careless responders may not carefully read and comprehend items or may be interrupted during completion and never complete the survey. Fraudulent responses may arise due automated responses due to ‘bots’. This may create bias because means and correlations are attenuated, measure reliability decreases, validity decreases, and the risk of Type 2 error increases (50). Of the 178 individuals who responded to the survey, 12 participants were excluded for not meeting the study inclusionary criteria, 6 for outlier response times, and 11 for missing substantial data, leaving a final analytic sample of 149.

Participants were recruited from online social media platforms (e.g., Facebook, Twitter, Reddit) and community-based organizations that serve TGNC POC adults within the U.S. (e.g., Queer and Transgender Network through The American Association for Marriage and Family Therapy, LGBTQIA+ health centers, etc.). Study flyers were posted to social media sources (e.g., Twitter, Instagram, etc.) by the research team with hashtags to gain visibility (e.g., #transgender). To recruit from private or closed online groups (i.e., Reddit, Facebook, etc.), the group administrator was contacted to obtain permission to recruit. Following permission, information about the study including the study flyer, IRB approval information, and contact information was provided to the administrator who then shared the information with potential participants or allowed the research team to share the information. For in-person organizations (e.g., health centers), the flyer was requested to be posted within the organization following approval.

### 2.2 Data collection

Prospective participants followed the survey link presented in the recruitment materials to the online Qualtrics survey (Qualtrics, Provo, UT) where they were presented with the recruitment statement, the informed consent document that outlined risks of the study, benefits, compensation information, etc. Participants then consented to the study. Following consent, participants answered screening questions and those who were eligible continued to the study survey, which included demographic questions, transition specific questions (i.e., gender marker, legal name change, GAC, etc.), and study related measures described below. To protect participant identity, responses were anonymized, and no personally identifiable information was collected. Upon survey completion, participants were thanked for their participation and directed to a separate Qualtrics survey to input their email address to receive compensation ($20 emailed gift-card) to aid in confidentiality. The survey took between 30 and 45 min to complete.

### 2.3 Measures and variables

Participants answered demographic questions (i.e., age, race, ethnicity, gender identity, assigned sex at birth, employment, sexual orientation, etc.) and transition-related questions about gender marker and legal name change: “Have you ever completed a legal name change to match your gender identity?” and “Have you ever completed a legal gender marker change to match your gender identity?” Answer choices included *yes*, *no*, or *I do not want*. Participants were asked about experiences with identification documents with, “When you have shown your ID with your name and/or gender marker that does not match your gender presentation, have any of the following occurred,” with participants able to select all of the following that apply: *I have been verbally harassed*, *I have been assaulted/attacked*, *I have been asked to leave*, *I have been denied services or benefits*, *I have been asked to a show a different form of ID*, *I have not experienced any of the above problems*, and *This does not apply to me because I have only shown IDs that match my gender identity*.

Participants were also asked about what types of GAC they ever wanted with the following study-developed question, “Have you ever wanted any of the health care listed below for your gender identity or gender transition?” Participants selected all that applied from the following choices: *counseling/therapy*, *hormone therapy/HRT*, *puberty blocking hormones*, *gender affirmative surgery* (i.e.*, top surgery, hysterectomy, phalloplasty, vaginoplasty, etc.*), or *none of the above*. If participants indicated they ever wanted any form of GAC from the previous question, they were presented, “Have you ever received any of the health care listed below for your gender identity or gender transition?” and selected all that applied from the same list as above. For participants that indicated they had received gender affirming hormone therapy, they were asked if they were currently using GAHT (*yes* or *no*). For the participants who were currently using GAHT, they answered, “Where are you currently getting your hormones from?” and could select all that apply from the following: *licensed professional* (i.e.*, doctor, endocrinologist, etc.*), *non-licensed sources* (i.e.*, internet*), and *friends or family*.

#### 2.3.1 Intersectional distal stressors

The LGBT People of Color Microaggressions Scale (53) measured intersectional distal stress. The scale measures how bothersome or distressing 18 items (e.g., “Difficulty finding friends who are LGBT from your racial/ethnic group,” “Being discriminated against by other LGBT people of color because of your race,” and “Being seen as a sex object by other LGBT people because of your race/ethnicity”) have been over the past 12 months. Participants select answers to the 18 items that range from 0 (it did not happen to me/not applicable) to 5 (it happened, and it bothered me EXTREMELY). Scores were calculated by computing the mean score across the measure, with higher scores indicating higher levels of average intersectional distal stressor distress (53).

The LGBT People of Color Microaggressions Scale has demonstrated construct validity and good internal consistency, *α* ≥ 0.81 for each subscale and *α* = 0.92 in the initial validation study (53). Supporting evidence also comes from a recent study with LGBTQ POC, including 31% transgender adolescents, which reported good reliability across subscales, reporting *α* = 0.81 for racism in dating and close relationships; α = 0.81 for heterosexism in racial-ethnic minority communities; α = 0.87 for racism in LGBTQ+ communities (54). Internal consistency in the current study was excellent (α = 0.92).

#### 2.3.2 Proximal stressors

The Gender Minority Stress and Resilience Measure (GMSRM) (55) was used to measure gender identity proximal stressors via internalized transphobia, negative expectations for the future, and nondisclosure of identity. The scale includes 22 items that measure level of agreement on a four-point scale (0 = strongly disagree to 4 = strongly agree), with higher scores indicating higher proximal stress. Proximal subscales items include: internalized transphobia, comprising eight items (e.g., “I resent my gender identity or expression” and “I feel that my gender identity or expression is embarrassing”), concealment, which includes five items (e.g., “Because I do not want others to know my gender identity/history, I modify my way of speaking”), and negative expectation for the future, including 9 items (e.g., “If I express my gender history, others would not accept me” and “If I express my gender history, I could be a victim of crime or violence”). The 22 items were summed to create a composite score, with total proximal stressor scores ranging from 0 to 88.

The GMSRM has demonstrated good criterion, convergent, and discriminant validity (55). Internal consistency was good in the validation study, with *α* = 0.91 for internalized transphobia, 0.89 for negative expectations for the future, and 0.80 for nondisclosure (55). The GMSR has continued to demonstrate strong reliability with BIPOC samples, with internal consistency ranging from *α* = 0.82 to 0.85. (56, 57). Internal consistency in the current study was good for overall proximal stressor scale (α = 0.88).

#### 2.3.3 Depression

The Patient Health Questionnaire-9 (PHQ-9) (58) was used to measure depression. The PHQ-9 is a nine-item measure of the severity of depression over the last two weeks (58). Participants indicated how often they have been bothered by nine problems (e.g., “Feeling down, depressed, or hopeless,” and “Poor appetite or overeating”) on a four-point scale (0 = not at all, 1 = several days, 2 = more than half the days, 3 = nearly every day). Scores from each item on the PHQ-9 were summed to create a composite score ranging from 0 to 27, with higher scores indicating higher levels of depression. The PHQ-9 is a widely used measure with strong psychometric properties, including strong reliability (*α* = 0.89) and criterion validity (0.88 for sensitivity and specificity) (58). Reliability with the TGNC population has also been strong ranging from α = 0.89 to α = 0.93 (59, 60). Internal consistency in the present study was good (α = 0.88).

#### 2.3.4 Anxiety

The Generalized Anxiety Disorder-7 (GAD-7) (61) was used to measure anxiety due to its previous use with transgender samples (59, 62). The GAD-7 measures the frequency that participants have been bothered by seven problems (e.g., “Feeling nervous, anxious or on edge”) over the past two weeks on a four-point Likert scale (0 = not at all, 1 = several days, 2 = more than half the days, 3 = nearly every day). Scores from each item were summed, to give a total composite score ranging from 0 to 21, with higher scores indicating higher anxiety (61). The GAD-7 had excellent internal reliability in the initial validation study (*α* = 0.92) (61), with transgender participants (α = 0.91) (59), and POC transgender participants (α = 0.92) (63). Internal consistency in the present study was good (α = 0.85).

#### 2.3.5 Gender congruence

The Gender Congruence Factor from the Gender Congruence and Life Satisfaction Scale (GCLS) (64) was used to measure gender congruence. The Gender Congruence Factor contains 17 items across four subscales that measure genitalia congruence, chest congruence, other secondary sex characteristics congruence, and social gender role recognition. Participants indicated the frequency (never, rarely, sometimes, often, or always) of occurrence in the last six months for each of the 17-items (e.g., “I have felt distressed when touching my genitals as they do not match my gender identity” and “I have felt that my body hair conflicts with my gender identity, either because I have it and do not like it or because I would like to have it”). Items are scored on a 5-point scale (1 = always to 5 = never), with higher scores indicating higher gender congruence. The mean score from the Gender Congruence Factor was calculated as the total gender congruence score.

The Gender Congruence Factor was selected due to its development for both transgender and nonbinary adults, good psychometric properties that include internal consistency of 0.77 or higher for each subscale of the factor, good test–retest reliability (0.84), and good discriminate validity for those who have medically transitioned and those who have not (64). The GCLS has demonstrated acceptable reliability outside of the validation study, with internal consistency ranging from *α* = 0.77 (25) to 0.86 (65). Internal consistency of the Gender Congruence Factor in the current study was poor (α = 0.42), with a wide range among subscales (0.58 genital, 0.24 chest, 0.52 secondary sex characteristics, 0.26 social gender role recognition).

### 2.4 Data analysis

All analyses were conducted at a two-tailed alpha of 0.05. Initial exploratory data analyses were conducted using IBM SPSS Statistics (Version 29). Overall descriptive analyses presented categorical variables as counts (n) and frequencies (%) and continuous variables as mean and standard deviation (SD) (Table 1). All outcomes (anxiety, depression, gender congruence), the predictor (intersectional distal stress), and the mediator (proximal stress) were normally distributed as evidenced by acceptable skewness (between −1 and 1) and kurtosis values below 2 (66). Pearson’s correlation analyses were used to assess the relationship among outcomes, predictor, and the mediator (Table 2). Bivariate associations between each outcome and potential control variables were assessed using independent samples t-tests or one-way ANOVAs (Table 3). No post-hoc tests were run for univariate ANOVAs, as the omnibus test was of importance in deciding which control factors to include in SEM models.

**Table 1:** Pearson correlation of independent, mediating, moderating and outcome variables.

Variable	Depression	Anxiety	Gender congruence	Distal stress	Proximal stress
Depression	1.00				
Anxiety	0.85***	1.00			
Gender congruence	−0.28**	−0.23*	1.00		
Distal stress	0.57***	0.58***	−0.25*	1.00	
Proximal stress	0.49***	0.54***	−0.38***	0.46***	1.00
Mean (±sd)	8.7 (±5.2)	7.0 (±4.0)	3.1 (±0.3)	2.1 (±0.7)	49.6 (±13.2)

*p < 0.05.

**p < 0.01.

***p < 0.001.

**Table 2:** Crude, bivariate relationship of proposed model covariates with outcomes, Pearson correlation, independent sample t-test or one-way ANOVA.

Variable	Depression		Anxiety		Gender congruence	
	r or m (±sd)	*p*-value	r or m (±sd)	*p*-value	rr or m (±sd)	*p*-value
Age	−0.06	0.499	−0.04	0.688	−0.09	0.333
Income		**0.028**		0.095		0.684
< $40,000	10.09 (4.62)		6.86 (4.45)		3.11 (0.29)	
$40,000–$60,000	7.29 (4.4)		6.48 (3.42)		3.17 (0.27)	
> $60,000	9.89 (6.54)		8.34 (4.66)		3.14 (0.27)	
Gender identity		**0.002**		**<0.001**		0.091
Woman/transfem	8.49 (5.00)		6.11 (3.98)		3.16 (0.27)	
Man/transmasc	7.96 (5.74)		6.69 (4.14)		3.19 (0.22)	
Other	11.83 (4.59)		9.64 (3.89)		3.06 (0.31)	
Name change		0.773		0.880		0.594
Yes	9.67 (5.66)		7.49 (4.61)		3.12 (0.27)	
No	9.38 (4.94)		7.37 (3.94)		3.15 (0.28)	
Gender marker		0.226		0.825		0.453
Yes	9.73 (5.57)		7.31 (4.58)		3.12 (0.26)	
No	8.59 (5.22)		7.15 (3.95)		3.16 (0.28)	

*r = Pearson correlation; sd = standard deviation.Bolded values indicate significant values, *p* <.05.

**Table 3:** Demographic, mental health, and stressor characteristics of participants (*n* = 149).

Variable	% (*n*) or mean (±sd)
Age: Mean (SD)	27.19 (±5.31)
Race: *n* (%)	
Black or African American	127 (86.4%)
White	10 (6.8%)
American Indian or Alaska Native	7 (4.8%)
Asian Indian	2 (1.4%)
Some other race	1 (0.7%)
Ethnicity: *n* (%)	
Hispanic or Latinx	18 (12.2%)
Not Hispanic or Latinx	130 (87.8%)
Gender Identity: n (%)	
Woman or transfeminine	56 (37.8%)
Man or transmasculine	53 (35.8%)
Other (Nonbinary, Genderqueer, Gender Questioning, Gender Nonconforming, Agender, Mahu, Muxe, Two-Spirit)	39 (26.4%)
Sex assigned at birth (*n* = 148): *n* (%)	
Male	88 (59.5%)
Female	58 (39.2%)
Intersex	1 (0.7%)
Prefer not to say	1 (0.7%)
Sexual orientation: *n* (%)	
Heterosexual or straight	54 (36.2%)
Gay or lesbian	51 (34.2%)
Bisexual or pansexual	52 (34.9%)
Other (Queer, Omnisexual, Asexual, Aromantic, Demisexual, Questioning, Sexually Fluid, Other)	41 (27.4%)
Prefer not to answer	2 (1.3%)
Employment status: *n* (%)	
Currently employed full-time	90 (61.2%)
Currently employed part-time	49 (33.3%)
Currently employed but not working	1 (0.7%)
Unemployed (all)	7 (4.8%)
Highest level of education completed (*n* = 149)	
Less than HS grad	5 (3.4%)
HS Degree or equivalent	24 (16.1%)
Vocational or technical program/training	21 (14.1%)
Some college	40 (26.8%)
Associate degree	15 (28.2%)
Bachelor’s degree	42 (28.2%)
Master’s degree or higher	2 (1.3%)
Annual individual income (USD): *n* (%)	
< $40,000 per year	50 (34%)
$40,000 - $60,000 per year	45 (30.6%)
> $60,000 per year	52 (35.4%)
Relationship status: *n* (%)	
In a relationship	45 (30.2%)
Divorced	4 (2.7%)
Civil Union/domestic partnership	7 (4.7%)
Married	37 (24.8%)
Separated	2 (1.3%)
Single (not looking)	14 (9.4%)
Single (casually dating)	40 (26.8%)
Widowed	1 (0.7%)
Prefer not to answer	1 (0.7%)
Relationship type: *n* (%)	
In a monogamous relationship	60 (41.2%)
In a non-monogamous or polyamorous relationship(s)	56 (39.2%)
Not in a relationship	26 (18.2%)
Legal name change *n* (%)	
Yes	81 (54.4%)
No	55 (36.9%)
Do not want	10 (6.7%)
Missing	3 (2.0%)
Legal gender marker change *n* (%)	
Yes	78 (52.3%)
No	68 (45.6%)
Do not want	1 (0.7%)
Missing	2 (1.3%)
Outcomes of showing ID with incongruent name or gender marker *n* (%)	
Verbal harassment	83 (55.75%)
Assault	44 (29.5%)
Asked to leave	55 (36.9%)
Denied services or benefits	44 (29.5%)
Asked to show a different ID	46 (30.9%)
None of the above	17 (11.4%)
Not applicable (Matching ID)	8 (5.4%)
Wanted gender affirming care (GAC): *n* (%)	
Counseling/therapy	84 (56.4%)
HRT	55 (36.9%)
Puberty blocking hormones (during youth or adolescence)	27 (18.1%)
Gender affirmative surgery	27 (18.1%)
Did not want	17 (11.4%)
Ever received GAC	
Counseling/therapy	96 (64.4%)
HRT	43 (28.9%)
Puberty blocking hormones (during youth or adolescence)	17 (11.4%)
Gender affirmative surgery	22 (14.8%)
None of the above	5 (3.4%)
Current hormone replacement therapy (HRT Use): *n* (%)	
Yes	31 (20.8%)
No	11 (7.4%)
Missing	107 (71.8%)
Age start HRT (*n* = 40): Mean (SD)	18.98 (±4.20)
Age of awareness of incongruence (*n* = 144): Mean (SD)	16.13 (±3.79)
Age of identity to others as TGNC (*n* = 131)	18.91 (±3.67)
Providers of HRT	
Licensed professional	27 (18.1%)
Unlicensed source (i.e., internet)	4 (2.7%)
Friends or family	1 (0.7%)
Anxiety (GAD-7) (*n* = 133)	7.45 (±4.27)
Depression (PHQ-9) (*n* = 135)	9.19 (±5.38)
Gender congruence mean (GCLS) *n* = 113	3.14 (±0.27)
Proximal stressors (GMSRM) *n* = 111	48.41 (±14.61)
Distal stressors (LGBT POC Microaggressions) *n* = 130	2.12 (±0.72)

To assess the direct and indirect effects of proximal stressors on the relationship of intersectional distal stressors and outcomes, a path analysis using structural equation modeling (SEM) framework was conducted with MPlus v8.8 (67). SEM permits the exploration of total, direct, and indirect effects of variables simultaneously. Bootstrapping analysis with 5,000 resamples was used in all analyses, as it is preferred over the Sobel test for testing indirect effects as it lessens the importance of multivariate normality assumptions (68). Missing data in MPlus were handled using full information maximum likelihood, which uses all available information in analyses. A saturated model was specified to estimate all direct and indirect paths among study variables; therefore, fit indices are not informative due to the zero degrees of freedom in saturated models (69). Our goal was to describe all relationships, as this was an exploration of possible direct and indirect effects and their magnitude. This analysis serves as a theoretical baseline, for larger, more robust samples and analyses. Total, indirect, and direct effects are shown as standardized beta coefficients (*β*) and 95% confidence intervals. Standardized beta coefficients were presented as they put all effects on a common scale (e.g., standard deviation), thereby, allowing comparison of the relative strength of predictors that may be measured in different units or scales. Multicollinearity was assessed with the variance inflation factor (VIF) and it was found that all variables had VIF < 5, indicating no multicollinearity.

## 3 Results

A total of 149 responses were included in this analysis (Table 1). The mean respondent age was 27.19 years (SD = 5.31). 86.4% of respondents identified as Black or African American and 12.4% identified as Hispanic or Latinx. While the high percentage of Black or African American participants limits the ability to generalize findings to other TGNC POC groups, it represents an opportunity to explore more insight into the experience of Black or African TGNC individuals. The majority of respondents (59.5%) were assigned male at birth, and the current gender identity breakdown of respondents was as follows: 37.8% Woman or Transfeminine; 35.8% Man or Transmasculine; and 26.4% any other gender. The average age of awareness of gender incongruence was 16.13 years (SD = 3.79) and average age of beginning to identify as such to others was 18.91 years (SD = 3.67).

More than half of respondents (57.7%) had earned an associate’s degree or higher and 94.5% were employed at least part-time with 61.4% being employed full-time. One third of respondents (34.0%) reported an individual annual income of less than $40,000 per year. Nearly two-thirds of respondents (59.7%) reported being in a committed relationship, with 39.3% reporting that relationship as non-monogamous or polyamorous. Mean scores on the PHQ-9 and GAD-7 were both in the “mild” range, 9.19 (SD = 5.38) and 7.45 (SD = 4.27) respectively.

### 3.1 Transition-related results

Nearly the whole sample reported a desire to obtain a legal name change (*n* = 136, 91.3%); however, only 59.6% (*n* = 81) of those who wanted a name change reported completing one. More participants desired a gender marker change (*n* = 146, 98%) but only 53.4% (*n* = 78) reported completing one. The most common outcomes of showing IDs that were incongruent with participant name and/or gender marker was verbal harassment (*n* = 83, 55.75%), being asked to leave (*n* = 55, 36.9%), and being asked to show a different ID (*n* = 46, 30.9%). Nearly 30% of participants (*n* = 44) reported assault as an outcome of ID incongruence. Only 17 participants (11.4%) reported not experiencing a listed negative outcome of ID incongruence.

Approximately 40% of participants desired ever wanting GAHT (*n* = 55) and 78.2% of those who desired GAHT reported receiving GAHT (*n* = 43). Of the 43 who ever received GAHT, 31 (72.1%) reported current use and most received GAHT from licensed professionals (84.4% of those receiving GAHT). For those who desired GAHT but were not currently using GAHT, the most common barriers cited were lack of resources (insurance and/or financial resources, *n* = 7), pressure from romantic partner or family members (*n* = 6), fear of harassment, discrimination, or violence (*n* = 5), and pressure from employer (*n* = 2). One participant reported that they realized gender transition was not right for them. Fewer participants desired GAC surgery than GAHT, with 18.1% (*n* = 27) reporting wanting surgery and 81.5% of those who wanted surgery received it (*n* = 22). 17 participants (*n* = 11.4%) reported not wanting any of the GAC sources listed.

### 3.2 Main analyses

**Figure 1:**
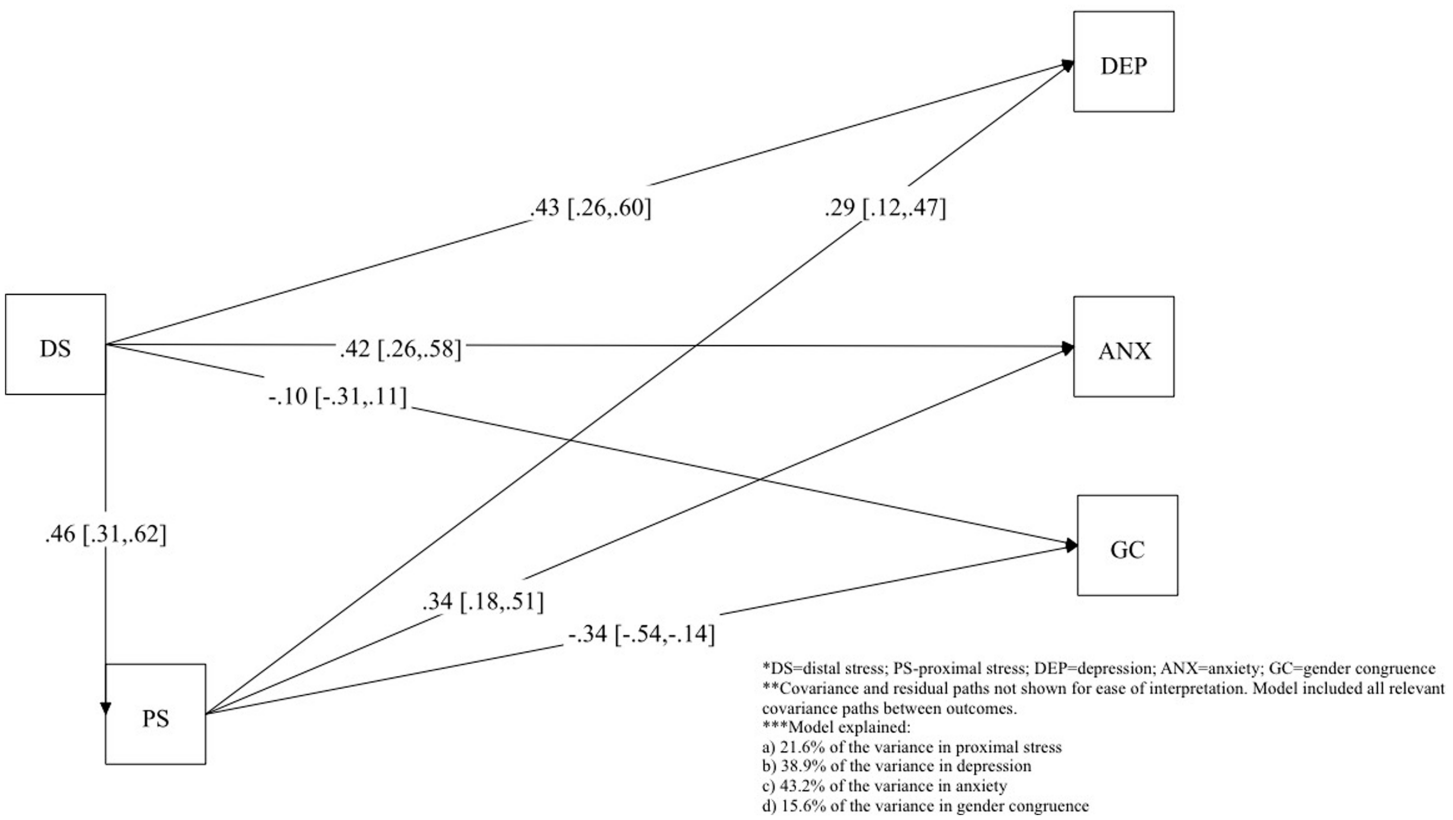
Saturated path model. DS, distal stress; PS, proximal stress; DEP, depression; ANX, anxiety; GC, gender congruence. Covariance and residual paths not shown for ease of interpretation. Model included all relevant covariance paths between outcomes. Model explained: (a) 21.6% of the variance in proximal stress; (b) 38.9% of the variance in depression; (c) 43.2% of the variance in anxiety; (d) 15.6% of the variance in gender congruence.

Final SEM results are provided in Table 4 (direct, indirect, total effects) and Figure 1 (final Path model with standardized coefficients). Distal stressors had significant positive direct and indirect effects, through proximal stressors, on depression and anxiety. Distal stressors had a significant positive direct (*β* = 0.43, 95% CI = 0.26, 0.60) and indirect effect via proximal stressors (*β* = 0.14, 95% CI = 0.04, 0.23) on depression. A similar pattern was found for the direct (*β* = 0.42, 95% CI = 0.26, 0.58) and indirect (*β* = 0.16, 95% CI = 0.07, 0.26) effect of distal stressors on anxiety. It was found that distal stressors did not have a significant direct effect on gender congruence, but a significant indirect effect through proximal stressors (indirect effect: *β* = −0.16, 95% CI = −0.27, −0.05). Figure 1 shows that the model explained 38.9% of the variance in depression, 43.2% of the variance in anxiety, and 15.6% of the variance in gender congruence. Finally, distal stressors had the strongest total effects on anxiety (*β* = 0.58, 95% CI = 0.45, 0.72), closely followed by depression (*β* = 0.57, 95% CI = 0.43, 0.70) and gender congruence (*β* = −0.26, 95% CI = −0.44, −0.07).

**Table 4:** Saturated model, direct, indirect and total effects of stressors on mental health and gender congruence.

Outcome and pathways	Direct effect β (95% CI)	Indirect effect via proximal stressors β (95% CI)	Total effect β (95% CI)
Proximal stressors			
Intersectional distal stressors	**0.46 (0.31, 0.62)**	–	**0.46 (0.31, 0.62)**
Depression			
Intersectional distal stressors	**0.43 (0.26, 0.60)**	**0.14 (0.04, 0.23)**	**0.57 (0.43, 0.70)**
Proximal stressors	**0.29 (0.12, 0.47)**	–	**0.29 (0.12, 0.47)**
Anxiety			
Distal stressors	**0.42 (0.26, 0.58)**	**0.16 (0.07, 0.26)**	**0.58 (0.45, 0.72)**
Proximal stressors	**0.35 (0.18, 0.51)**	–	**0.35 (0.18, 0.51)**
Gender congruence			
Intersectional distal stressors	−0.10 (−0.31, 0.11)	**−0.16 (−0.27, −0.05)**	**−0.26 (−0.44, −0.07)**
Proximal stressors	**−0.34 (−0.54, −0.14)**	–	**−0.34 (−0.54, −0.14)**

Standardized beta coefficients and 95% confidence intervals (*n* = 94).Bolded values indicate significant beta coefficients.

## 4 Discussion

Following intersectional critiques from the literature, this study sought to explore the utility of the Gender Minority Stress Model with TGNC POC adults in the U.S. Given most of the literature in this area has predominantly been conducted with White TGNC, our study extends the research and demonstrates the applicability of the Gender Minority Stress Model to TGNC POC adults when measuring intersectional distal stressors. In alignment with our first aim, we found intersectional distal stressors were related to higher levels of anxiety and depression, both directly and indirectly through proximal stressors, suggesting intersectional distal stress may be related to mental health both directly and as a result of internalization of those experiences. Similar findings were demonstrated when using gender minority distal stressors within the Gender Minority Stress Model for psychological distress (63) and depression (70). Prior research found TGNC POC experience higher rates of distal stressors including discrimination and violence as compared to their white TGNC peers, which is associated with higher rates of suicidal ideation, suicide attempts, self-harm, and foregone physical health care for this population (71). TGNC individuals often experience misgendering, a form of nonaffirmation and distal stress that may be intentional or microaggressive, which is related to gender dysphoria and adverse mental health outcomes (72, 73). Misgendering is more likely to occur for TGNC POC than White TGNC peers (74).

Contrary to our prediction, intersectional distal stress was not directly associated with gender congruence and instead was fully indirectly associated with gender congruence through proximal stressors. This is similar to previous multiracial studies finding TGNC individuals internalize narratives (e.g., internalized shame and hypervigilance for threats to safety) as explanations for distal stressors that heightened gender incongruence (i.e., dysphoria) (75). Hypervigilance and race-based traumatic stress occur for POC people in the United States due to racism - interpersonal and structural - creating constant threats to their health and safety (76). For TGNC POC people, hypervigilance includes monitoring for racialized sexual objectification for avoiding victimization (77). In this way, our study adds to research on TGNC POC people demonstrating the effect of multiple marginalizations on their health and wellbeing (78). Within the literature, microaggressions for TGNC individuals are related to both gender incongruence (79) and mental health outcomes (e.g., anxiety and depression) (80–84). While our findings fit with these highlighted studies, the results may also be a product of the low reliability of the measure used for gender congruence making true effects hard to find or overestimating effects of other variables in the model.

Previous research using the Gender Minority Stress Model primarily explored gender incongruence through gender dysphoria as a proximal stressor (39, 83), finding preliminary support. On the contrary, another study found gender dysphoria fully mediated only the relationship between minority stress and eating pathology but did not add any additional variance for anxiety and depression (84). This suggests gender dysphoria may also serve as an outcome variable within the model, which our study sought to explore. In a recent systematic review, the additional variable of body image was highlighted because it predicts gender dysphoria and disordered eating across treatment case studies and empirical research (85). Leading treatment for eating disorders for TGNC people includes GAC, GAHT, and social transition elements for improving body image and reducing the use of eating patterns for changing body shape and size (85). This differs from previous models, which found gender congruence mediates distal stressors, explaining the association with psychological distress (65) and that distal stress is indirectly associated with psychological distress through gender dysphoria (86).

Despite the conceptualization as a proximal stressor within the Gender Minority Stress Model in the literature, few studies to date have explored the impact of both distal and proximal stress on gender incongruence, with some measuring only proximal stress (83) and others combining distal and proximal into one composite measure of minority stress (84). Our study highlights the importance of considering both racial and gender identity minority stressors and the association of those stressors with gender congruence. While our understanding of gender congruence has grown in recent years (26–87), these understandings have been primarily shaped by quantitative studies of White TGNC individuals (20, 25, 39) and limited qualitative studies of TGNC POC (e.g., 41, 88, 89). Thus, our findings contribute to the literature on gender congruence through the exploration of the association of proximal and intersectional distal stressors on gender congruence with an understudied group within the TGNC population.

Our study contributes to the literature on gender transition experiences for TGNC POC adults within the U.S. We found TGNC POC participants faced barriers to medical and social gender transition. While most of those who desired GAHT were able to obtain it, those who could not reported a lack of financial and insurance resources, pressure from their romantic partners or family members, fears of transgender related distal stress, and pressure from their employers as barriers. Although these are common barriers for all TGNC people to GAHT, the pressures from romantic partners and family members may also be connected to the unique, racialized fears of many Black and Latine/Hispanic families with TGNC youth. Black and Latine/Hispanic families already describe fears for their children’s futures, safety, and health given racism and xenophobia in the US (90, 91); the addition of a TGNC child may create additive fears that the life of this young person is only going to be made even harder if they go forward with a gender transition (92). It is possible this may lead to efforts on the part of the family to not support GAHT or other gender transition steps.

Regarding social transition, nearly all of the participants in this study desired access to identification documents aligned with their gender identity; however, between 50 and 60% of participants were unable to complete a legal name or gender marker change for these documents. Obtaining a gender marker change on documents can be more challenging than a legal name change in some states in the U.S. due to restrictive policies, inaccessible forms, and numerous and complicated steps and processes for applicants (93). As a result of mismatched identification, participants reported experiencing verbal harassment, being asked to leave or show another form of identification, and experiencing assault. Obtaining GAC when desired is essential for mitigating distal stressors, as TGNC individuals receiving GAC report lower levels of anxiety, depression, gender dysphoria (21, 22) and improves treatment outcomes for eating disorders (85).

Desire for GAHT in our sample was 40%, lower than findings from the U.S. 2022 Transgender Survey (88%) (94), which may suggest differences in transition-related goals for TGNC POC adults and reflect a growing understanding that gender incongruence may be due to distress related to one’s body (physical gender dysphoria) (64), distress due to the incongruence between one’s internal sense of gender and their embodiment or social experience (mental dysphoria) (26), and a misalignment between one’s gender identity and social environment (social dysphoria) (24). Within the limited body of literature, TGNC POC describe additional themes related to gender dysphoria, such as cultural gender ideals and self-harm, in addition to the themes endorsed by largely White TGNC samples experiences of bodily disconnection, emotional distress, and changes over time (28).

### 4.1 Clinical implications

The findings of the current study highlight the importance of culturally-competent, gender-affirming, individualized, and accessible healthcare and mental health services for TGNC POC. In particular, the findings highlight the significance of intersectional distal stressors and internalized stressors on the mental health of TGNC POC. Our study demonstrates there is a gap in how many participants wanted GAC and how many participants were able to access it. While systemic barriers such as insurance coverage, cost, policies banning gender-affirming medical care by some hospital systems, and state-level legal restrictions on GAC are related to these barriers, the literature also suggests that prior negative experiences in the healthcare system (e.g., misgendering by medical professional, deadnaming, lack of provider knowledge on transgender healthcare) and medical mistrust for POC communities (42, 95) also relate to these disparities (18). Providers may contribute to these negative experiences through assumptions of transition-related goals for TGNC POC adults. Current clinical interventions have primarily focused on facilitating medical transition (14, 35, 96) or were developed for TGNC youth (97, 98), highlighting the importance of providers exploring and identifying individualized gender transitions plans, barriers to accessing needed medical care, and internalized stressors given this study’s findings.

When clinicians are providing care to TGNC POC individuals, a biopsychosocial-spiritual and culturally humble approach (99) to addressing their concerns, especially those related to anxiety, depression, and gender dysphoria, should be considered. Our findings also highlight the need for targeted psychotherapeutic approaches to focus on reducing the internalization of shame and transphobia to facilitate gender transition, whether through social, mental, or medical avenues. This is particularly relevant, given the GAC access barriers due to legislation may require extra steps for locating a provider and travel (100).

Clinicians should be aware of the limitations of common screening tools to assess mental health pathology in this population, as the results may not capture the full extent of a TGNC POC patient’s experience with mental health and gender dysphoria. Racial and gender biases can influence diagnostic accuracy, leading to greater validity for certain groups over others, as seen in conditions like mood disorders and autism spectrum disorder (101). Given that symptom expression can vary across populations, current measures of gender congruence or dysphoria may be more valid for White TGNC individuals than for those from other racial or ethnic backgrounds. To reduce diagnostic bias, the use of structured mental health screenings, self-report measures (including psychological tests), structured interviews, and statistical prediction tools has been recommended (101).

### 4.2 Policy implications

Our study, and others like it, make plain that the mental health of TGNC POC people is supported by a reduction in intersectional distal stressors at the interpersonal, economic, and structural policy and legal levels. The current U.S. sociopolitical landscape and efforts at the federal and state level, are creating increasing policy and legal barriers to GAC, gender marker changes, legal name changes, and the like for TGNC people. In addition, economic disparities continue to widen between POC populations and White populations in the U.S. (102).

As of September 2025, 27 US states have enacted policies to restrict youth access to gender affirming care, with eight of those states also having policies restricting GAC for adults (103). Many of the policies affecting access to care for adults specifically prohibit Medicaid coverage for any GAC and have removed requirements from private health insurance plans to cover GAC. Some states have enacted laws regarding non-medical transition as well, such as South Carolina’s ban on public school staff using a child’s name or pronouns if they are different from what they were assigned at birth (104). Four U.S. states do not allow for the updating of a driver’s license gender marker under any circumstance, and 10 states require proof of surgery, court order, or amended birth certificate. Eight states do not allow the amending of the gender marker on birth certificates, eight states require both a court order and proof of surgery, and three states require proof of surgery only (93). At the federal level, the current U.S. administration crafted executive orders defining gender on the binary, calling gender affirmation of TGNC people “gender ideology extremism,” and defining GAC as “mutilation” of TGNC youth (105). This has led to changes on federal websites for mental and medical healthcare warning against “gender ideology” as well as the disappearance of federal datasets that include TGNC participants. None of the executive orders cite empirical research or leading medical (e.g., American Medical Association) and mental health (e.g., American Psychological Association, American Association of Marital and Family Therapy) associations who provide affirming guidelines for their membership (105). TGNC POC are differentially impacted by anti-TGNC legislation and policies due to several factors, including additional structural and interpersonal racism, disparities in economic resources, and the increased likelihood of living within states with those policies (106, 107).

In the current political landscape, it is already incredibly difficult for TGNC people to undergo any degree of medical or social transition, particularly TGNC POC. Our study shows participants experienced barriers to navigating the legal process of name and/or gender marker change. Many participants who were unable to do so may have been concerned for their safety during circumstances in which their legal documents do not match their gender expression, such as during routine traffic stops or while traveling. The increasing restrictiveness of U.S. policies surrounding medical, legal, and social transition is likely to increase hypervigilance, anxiety, gender dysphoria, and internalized stigma among TGNC people, with TGNC POC being especially at risk (108). Future research on Gender Minority Stress Models with POC samples will need to include state level indicators of TGNC equality, like those created by MAP (109), and racial and economic inequalities. This would allow researchers to see more clearly the context of participants for understanding the varied health outcomes.

### 4.3 Strengths and limitations

The current study had several notable strengths, such as the inclusion of both binary and nonbinary gender identities and the purposeful recruitment of POC participants. While the literature on gender identities outside of the binary has grown in the past few years, few studies including TGNC within nonbinary identity categories have been conducted. Given the higher rates of psychological distress experienced by nonbinary compared to binary individuals (110), further research is needed to understand unique impacts of race on gender identity.

In addition to the identified strengths, there are limitations of the current study. The first limitation is the low reliability of the GCLS (64) within our sample to measure gender congruence. We found large differences in the reliability of the different subscales within the measure. Other studies have highlighted challenges with accurately quantifying the experience of gender congruence across all demographic groups (29, 111). Specifically, the authors found low reliability (*α* = 0.37) for the social gender dysphoria subscale of the GCLS when used with a sample of 53.8% TGNC POC adults compared to White TGNC participants (29). They found that item 19, “I have found in distressing that others do not address me according to my gender identity” was not significantly related with other scale items, removing it from the analysis. After removal, Cronbach’s alpha improved to 0.59 for White participants and 0.40 for POC participants (total α = 0.50). The measure has been largely used with White TGNC adults [e.g., (65, 112)], suggesting the GLCS (64) may not adequately capture gender congruence for this population. Direct and indirect effects related to GCLS should be interpreted with caution, as the authors recognize that estimates may be unreliable in this context. However, considering this analysis was exploratory in a limited sample, results provide some initial evidence of the relationship of distal and proximal stressors on gender congruence. Future research could assess the reliability and validity of different gender congruence measures with a larger diverse TGNC sample.

Additionally, the study is limited by its cross-sectional nature. We cannot draw causal conclusions about the impact of distal and proximal stressors on anxiety, depression, and gender congruence across time. Further, our sample was mostly Black/African American TGNC, which limits the ability to generalize our findings to other TGNC POC groups. Future research should seek to understand the intersectional minority stressor experiences of other TGNC POC groups. To address challenges with temporality and intersectionality, The Temporal Intersectional Minority Stress (TIMS) Model has been proposed to situate minority stressor experiences within the sociocultural context in which they occur (113).

### 4.4 Future directions

Recent distal stressors including U.S. policies limiting access to GAC, bans on TGNC individuals in sports, and bathroom bills are associated with increases in anxiety and post-traumatic stress disorder (PTSD) (114). Future research is needed to further explore the differential impact of these policies, as TGNC POC may experience increased racial microaggressions due to U.S. policies focused on race related to immigration, crime, and gender minority stressors due to executive orders or changes in state laws reducing access to GAC. As a result, Latinx TGNC may be differentially affected, exacerbating existing feelings of gender incongruence, anxiety, and depression.

Future research should seek to develop measures that capture the experience of gender congruence and intersectional minority stressors for TGNC POC individuals. One new measure, the Multidimensional Gender Dysphoria Measure (MGDM) (111), was developed and validated with nearly equal numbers of White and TGNC POC participants, with varied gender identities present. The MGDM (111) highlighted the multidimensional nature of gender dysphoria, revealing five factors: (1) body gender dysphoria, (2) social gender dysphoria, (3) variability of gender dysphoria, (4) gender dysphoria relief, and (5) internalized gender normativity, offering an opportunity to continue exploring experiences of gender dysphoria with TGNC POC adults.

The current study used the LGBT People of Color Microaggressions Scale (53) as a proxy for intersectional distal stressors. Future research could use a recently updated version of the scale which better separates the conceptualization of heterosexism and cissexism for TGNC POC (115). In addition, researchers should seek to develop TGNC specific measures of intersectional distal stress, given the higher rates of distal stressors experienced by the TGNC community compared to other sexual identity minorities (116). Utilizing an intersectional lens allows further research into the experiences of TGNC POC who encounter both racism and transphobia (117).

Finally, research should focus on identifying protective factors, such as living in a state or city with special protections and policies for TGNC people and GAC. Protective factors in the community and close relationships can reduce the impact of minority stress on mental and physical health outcomes (118–120). Identifying additional protective factors may guide systemic and individual intervention development and move away from a deficit focused approach to research and clinical practice with TGNC POC. Given that focusing on gender dysphoria has been criticized as facilitating the medicalization of TGNC experiences (121), studying gender euphoria, the positive feelings and experiences related to gender congruence (122), represents one pathway toward celebrating TGNC identities that has been limited (123). Specifically, research should focus on gender euphoria for TGNC POC to fully understand their experiences intersectionally as TGNC and POC people (124).

## 5 Conclusion

The results of our study highlight the intersectional minority stressor experiences of TGNC POC within the U.S. and offer support for the applicability of the Gender Minority Stress Model to this population. Results revealed that the internalization of intersectional distal stressors were associated with lower levels of gender congruence through proximal stressors, while distal stressors were both directly and indirectly, through proximal stressors, associated with the mental health outcome. The internalization of negative messages about TGNC POC identities represents an area for clinical intervention, while intersectional distal stressors represent an area for policy intervention.

## Data Availability

The original contributions presented in the study are included in the article/supplementary material, further inquiries can be directed to the corresponding author.
